# Effectiveness of the KENKOJISEICHI local revitalization system on cognitive function change in older adults with mild cognitive impairment: study protocol for a randomized controlled trial

**DOI:** 10.1186/s13063-018-2642-3

**Published:** 2018-05-11

**Authors:** Songee Jung, Sungchul Lee, Seongryu Bae, Sangyoon Lee, Keitaro Makino, Yohei Shinkai, Hiroyuki Shimada

**Affiliations:** 1 Department of Preventive Gerontology, Center for Gerontology and Social Science, National Center for Geriatrics and Gerontology, 7-430 Morioka, Obu, Aichi 474-8511 Japan; 2 Japan Society for the Promotion of Science, 5-3-1 Kojimachi, Chiyoda, Tokyo, 102-8472 Japan

**Keywords:** Cognitive activity, Dementia, Older adult, Physical activity, Social activity, Outdoor behavior

## Abstract

**Background:**

Physical activity is associated with a lower risk of cognitive decline in older adults. However, past studies have demonstrated that older adults tend to relapse into inactivity after completing interventions. This ongoing study employs a randomized controlled trial design to assess the efficacy and efficiency of the KENKOJISEICHI local revitalization system for promoting daily outdoor behaviors to improve cognitive function in community-dwelling older adults with mild cognitive impairment (MCI).

**Methods/design:**

This 6-month randomized controlled trial will include 83 community-dwelling older adults aged 65 years or older with MCI. Participants will be randomized to the KENKOJISEICHI experimental group or an educational control group. The KENKOJISEICHI group will receive a 90-minute session twice per week that consists of social, intellectual, and physical activities involving outdoor behaviors intended to support cognitive function. Participants in the educational control group will attend two 120-minute educational classes during the 6-month trial period. Considering a 20–30% dropout rate, a sample size of 35 participants per group is required.

**Discussion:**

If the program successfully promotes long-term habitual outdoor behaviors, this will expand knowledge regarding how to support social, intellectual, and physical activities, as well as communication change, among the older population to provide them with cognitive benefits.

**Trial registration:**

University Hospital Medical Information Network (UMIN), Japan, UMIN000026479. Registered on 9 March 2017.

**Electronic supplementary material:**

The online version of this article (10.1186/s13063-018-2642-3) contains supplementary material, which is available to authorized users.

## Background

In mild cognitive impairment (MCI), several clinical and neuropsychological pathological features are present prior to the onset of overt Alzheimer’s disease [1]. Epidemiological data suggest that physical activity (PA) is associated with a lower risk of dementia [2], and many studies have identified the effects of exercise or PA on cognitive function in older adults with MCI [3–5]. Not surprisingly, there has been a sudden increase of interest in cognitive training and cognitive exercise interventions that can slow or reverse such changes or mitigate their detrimental effects [6].

Cognitive training and cognitive exercise interventions, involving guided practice of specific cognitive tasks, and cognitive stimulation programs, aimed at enhancing general cognitive functioning, have been offered to prevent or minimize the effects of cognitive aging. The effects of such cognitive interventions were investigated not only in healthy older adults but also in people with cognitive deterioration beyond normal age-dependent changes, such as people with MCI [6–8]. However, poor exercise adherence may be the most essential issue that hinders acquisition of health benefits and success of health promotion policies. Approximately 48% of older adults are reported to cease exercising within 6 months of initiation of an exercise program [9], subsequently leading to the loss of training effects [10].

Various studies have investigated factors that affect cognitive function and the development of dementia. In this context, the term social participation is used in a broad sense relating to society. Older adults with high levels of social participation are considered to have higher cognitive functioning than those with low levels of social participation [11–15]. In addition, social networks such as those related to work and volunteerism, as well as to friends and family, have been reported to exert a functional and protective effect with respect to dementia in older adults [14, 16]. Thus, it is possible that activity and cognitive function could be maintained by increasing social network involvement, such as with friends and family.

KENKOJISEICHI refers to a combination of health and self-employment, network development within one’s town, and a system of using neighborhood amenities such as stores, shops, cafes, supermarkets, libraries, community centers, and sports gyms in Takahama city, Aichi, Japan. We developed a structured program based on this concept, which combines physical, cognitive, and social activity promotion approaches with community resources for older adults. The program specifically focuses on helping participants to develop “continuous activities” based on enjoyment. These favorite activities include those that are physical, social, and intellectual, and involve participation in the community. Although such coordinated approaches have the potential to achieve substantial health promotion among the older population, they have not been adequately examined using randomized controlled trials. Therefore, the purpose of this study is to examine the effectiveness of the KENKOJISEICHI local revitalization system for promoting daily outdoor behaviors to improve cognitive function among community-dwelling older adults with MCI through a 6-month randomized controlled trial.

## Methods/design

### Design and setting

**Fig. 1:**
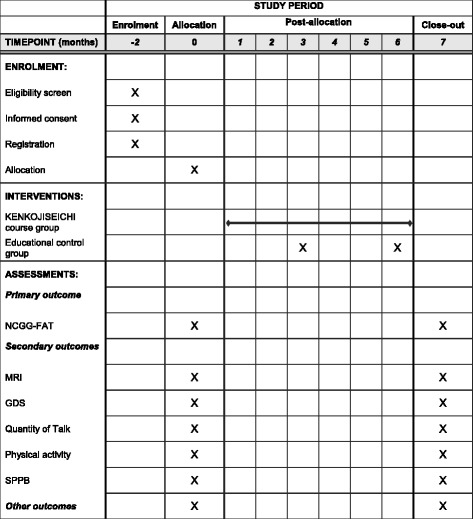
Intervention schedule of enrollment, interventions, and assessments

The study design is a randomized, single-blind, controlled trial. The 6-month-long study employs two groups: the KENKOJISEICHI course group and an educational control group. The study design and setting are shown in Fig. 1. This study will be conducted in Takahama city, Aichi, Japan. We have been conducting an observational study, including face-to-face interviews and measures of physical and cognitive function, in this community since 2015.

This study was conducted in accordance with the guidelines proposed in the Declaration of Helsinki, and the study protocol was reviewed and approved by the research ethics committee of the National Center for Geriatrics and Gerontology (NCGG), Japan (approval no. 861-2). The protocol was registered with the UMIN Clinical Trials Registry (UMIN000026479). We held a meeting for all potential participants to explain the study purpose, measurements, and design, as well as to obtain written informed consent.

### Participants

**Fig. 2:**
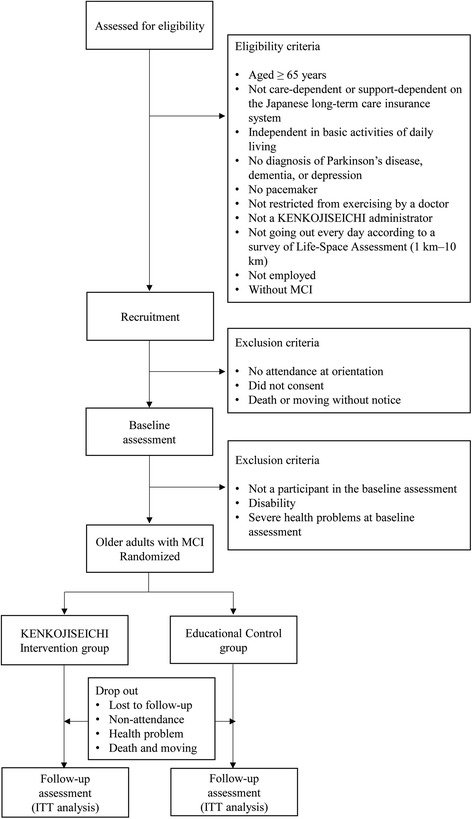
Flowchart of the study participants

A flow diagram of study participants is presented in Fig. 2. The study participants include community-dwelling older adults who were part of a subcohort of the National Center for Geriatrics and Gerontology–Study of Geriatrics Syndromes, conducted in Takahama from 2015 to 2016. The eligibility criteria were as follows: (1) aged 65 years or older; (2) not care-dependent or support-dependent on the Japanese long-term care insurance system; (3) independent in basic activities of daily living; (4) no diagnosis of Parkinson’s disease, dementia, or depression; (5) not restricted from exercising by a doctor; (6) no pacemaker; (7) not a KENKOJISEICHI administrator; (8) not going out every day according to the Life-Space Assessment (LSA; 1 km–10 km); (9) not employed; and (10) without MCI (amnestic MCI and nonamnestic MCI). Exclusion criteria were as follows: (1) no attendance at orientation, (2) consent not provided, (3) death or moving without notice, (4) not a participant in the preintervention assessment, (5) disability, and (6) severe health problems at baseline assessment. All of the participants read and signed the informed written consent form that was approved by the institutional review board for testing.

### Sample size

The required sample size for this study was calculated using predictions of 6-month changes in NCGG Functional Assessment Tool (NCGG-FAT) results. On the basis of results of our previous work, we predicted that a change of 2.0 points would indicate a difference [17, 18]. To detect such a difference, with a power of 80% and alpha error of 5%, and considering a 20–30% dropout rate, a sample size of 35 participants per group was required.

### Randomization

After the baseline assessment, participants will be randomly assigned to the KENKOJISEICHI group or an educational control group. A researcher who is unaware of the aims of the study will perform the randomization procedure using computer-generated random numbers and will send the assignments to a researcher. Then, this researcher will notify the participants by mail. They will be told that they can stop the intervention at any time. They will also be asked to maintain their usual activities and to not participate in other research projects related to exercise or physical interventions.

### Planned trial interventions

#### KENKOJISEICHI group

The KENKOJISEICHI group will participate in classes focusing on 101 outdoor behaviors, including 28 physical activities, 29 intellectual activities, and 44 social activities; a small group gathering meeting will also be available. Locations that meet at least two of the following five examination requirements will be certified as healthy places: (1) places where KENKOJISEICHI group members can participate as they choose and can carry out activities with a purpose, (2) places where they can participate lightly, (3) places where people can interact with other people in the community, (4) places where the chosen activity can be performed, and (5) places where relaxation and relief can be realized. Nevertheless, a site that is used for political or religious activities or for the purpose of promoting these activities will not be recognized for KENKOUJISECHI purposes.

Social activities will be conducted in a community network and will include events such as traditional and health festivals, as well as visiting a cafeteria, drug store, shopping mall, temple, sightseeing spot, senior’s club, or variety store. Cognitive activities will include visiting a library, singing karaoke, playing the Japanese game Go, reminiscing (life review), arts and crafts, doing calligraphy, video impression, creating stamp art, and visiting an art museum. Physical activities will include Tai Chi, machine studio, indoor golf, walking, muscle strength training, stretch, hula dance, traditional dance, boccia, and social dance.

The intervention program consists of participating in social (16 times), intellectual (16 times), and physical activities (16 times). The forty-eight 90-minute sessions take place twice per week over 24 weeks and include a 15-minute condition check and stretching; 60 minutes of social activity, intellectual activity, or PA; and 15 minutes to write a report, provide impressions, and discuss the upcoming schedule. Participants will be divided into nine groups with two to four participants and two to three staff members in each group. The staff will manage the schedule, check health during participation, and prepare reports. This program focuses on improving outdoor behaviors and communication; therefore, it was designed to be simple and performed at any location in order to facilitate long-term adherence for all participants.

Participants will choose their next KENKOJISEICHI location by talking with the staff and through group-based activities (Table 1). The participants will record their impressions about the content of each day’s activity. Then, staff will write a brief activity report and note feedback from participants.

**Table 1:** KENKOJISEICHI curriculum

	One time	Two time	Three time	Four time	Five time	Six time	Seven time	Eight time
A group	Social	Physical	Intellectual	Social	Physical	Intellectual	Social	Physical
B group	Physical	Intellectual	Social	Physical	Intellectual	Social	Physical	Intellectual
C group	Intellectual	Social	Physical	Intellectual	Social	Physical	Intellectual	Social
D group	Social	Physical	Intellectual	Social	Physical	Intellectual	Social	Physical
E group	Physical	Intellectual	Social	Physical	Intellectual	Social	Physical	Intellectual
F group	Intellectual	Social	Physical	Intellectual	Social	Physical	Intellectual	Social
G group	Social	Physical	Intellectual	Social	Physical	Intellectual	Social	Physical
H group	Physical	Intellectual	Social	Physical	Intellectual	Social	Physical	Intellectual
I group	Intellectual	Social	Physical	Intellectual	Social	Physical	Intellectual	Social

*Social* refers to social activity, *physical* refers to physical activity, and *intellectual* refers to intellectual activity

#### Educational control group

Participants in the educational control group will attend two 90-minute educational classes during the 6-month trial period. The classes will taught by experts on topics considered less likely to influence cognition and PA (e.g., nutrition, pain, and oral care).

### Data collection

Assessments will be conducted at baseline and at postintervention (Table 2). Assessment, data entry, and data analysis will be performed by a different researcher blind to group allocation and not involved in delivering the interventions.

**Table 2:** List of measurements collected at preintervention and postintervention assessments

Category		Measurement
Primary outcome		National Center for Geriatrics and Gerontology-Functional Assessment Tool (NCGG-FAT)
Secondary outcomes	Neuroimaging	Magnetic resonance imaging (whole-brain and hippocampal volume)
		Geriatric Depression Scale (GDS)
		Quantity of talk (Silmee W20 monitor)
	Physical function	Short Physical Performance Battery (SPPB; balance, side-by-side stand, semitandem, and tandem tests; gait speed; 2.4-m usual pace walk test; and lower extremity strength chair stand test)
		Trail walking test (TWT)
		Handgrip strength
	Physical activity	Triaxial accelerometer (modified GT40-020)
Other outcomes	Self-reported health status interview	International Physical Activity Questionnaire (IPAQ)
		Kihon Checklist (KCL)
		Life-Space Assessment (LSA)
		Pittsburgh Sleep Quality Index (PSQI)
		Short Nutritional Assessment Questionnaire (SNAQ)
		5-Item World Health Organization Well-Being Index (WHO-5)
		Japan Science and Technology Agency Index of Competence (JST-IC)
		Subjective memory complaints (SMCs)
		Hearing Handicap Inventory for the Elderly–Screening Version (HHIE-S)
	Health-related factors	Diseases diagnosed, prescribed medications, blood pressure, smoking status, alcohol intake, living-alone status, outing frequency, falls, fall-related injuries, fear of falling, urinary incontinence, habitual exercise, habitual activity, mobility limitation, hearing aids, vision, walking aids, pain, talking, talking time, outdoor activities, self-efficacy, outdoor social support, self-cognition, quality of life, basic activities of daily living (BADL)
	Body composition and anthropometry	Multifrequency bioelectrical impedance analysis (body fat and muscle volumes)
		Body weight and height

#### Primary outcome

The primary outcome measure is cognitive function at the 6-month postintervention assessment. Cognitive function will be assessed using the NCGG-FAT [19, 20], a multidimensional neurocognitive test, to assess memory (word list memory-I: immediate recognition; word list memory-II: delayed recall), attention and executive function (electronic tablet version of the Trail Making Test A and B), processing speed (electronic tablet version of the Digit Symbol Substitution Test), and visuospatial function (original version of the NCGG-FAT). High test-retest reliability and moderate to high validity were confirmed in community-dwelling older adults for all task components of the NCGG-FAT.

#### Secondary outcomes

Secondary outcomes will be brain and hippocampal volume, mental status, and depressive symptoms, which can potentially explain the mechanisms of the effects of the KENKOJISEICHI activities on the primary outcome. Whole-brain and hippocampal volume will be determined using a 3-T magnetic resonance imaging system (TM Trio; Siemens Healthcare, Erlangen, Germany). Three-dimensional volumetric acquisition of T1-weighted gradient-echo sequences will be performed to produce a gapless series of thin sagittal sections using a magnetization preparation rapid-acquisition gradient-echo sequence (inversion time [TI] 800 milliseconds, repetition time [TR] 1800 milliseconds, echo time [TE] 1.99 milliseconds, and 1.1-mm slice thickness). Axial T2-weighted spin-echo images (TR 4200 milliseconds, TE 89.0 milliseconds, and 5.0-mm slice thickness) and axial fluid-attenuated inversion recovery images (TI 2500 milliseconds, TR 9000 milliseconds, TE 100 milliseconds, and 5-mm slice thickness) will then be obtained for diagnosis.

Depressive symptoms will be measured using the Geriatric Depression Scale [21], which is a self-report screening tool developed to detect depression in older adults. The responses to the 15-item questionnaire will be scored, with scores of 0–4 defined as normal, 5–10 as mildly depressed, and 11–15 as severely depressed [22, 23]. A total score ≥ 5 points is considered to indicate depressive symptoms.

Quantity of talk will be measured using a biosensor device (Silmee™W20; TDK Co., Ltd., Chiba, Japan) for continuous monitoring of the volume of talk. All of the participants will be asked to wear the biosensor over their right wrist for 14 days following their examination while outdoors, except while swimming or bathing, and to maintain their usual activities.

PA will be measured using a triaxial accelerometer (modified HW-100; Kao Co., Ltd., Tokyo, Japan) for continuous monitoring of the intensity of PA. We will calculate parametric values, including the weekly average, for the daily amounts of light-intensity PA (1.5–2.9 metabolic equivalents [METs]) and moderate to vigorous PA (≥ 3.0 METs). The data will be recorded in 4-second epochs for up to 1 day and will be collected using the KAO system installed at the KENKOJISEICHI. The display of the accelerometer will be blinded. For inclusion in the present study, a valid day will be defined as a day in which the accelerometer is worn for ≥ 10 hours, with a total wear duration ≥ 7 days.

Participants in our study will perform three physical function tests. First, the Short Physical Performance Battery is used to measure balance, gait speed, and lower extremity strength [24]. The total score as well as each component score is related to the degree of disability, and these scores are strongly associated with mortality and hospital admission. Second, in the trail walking test, flags are installed randomly at each of 15 positions [25]. Third, handgrip strength will be measured using a handheld dynamometer (T.K.K. 5401 GRIP D; Takei Scientific Instruments, Tokyo, Japan), with participants in a standing position with their arms hanging naturally at their sides [26].

#### Other outcomes

The participants will complete a self-report health status interview to provide information on a variety of health-related factors. PA will be measured using the International Physical Activity Questionnaire [27]. Low, moderate, and high levels of PA will be calculated on the basis of METs for different activities. Basic health will be measured using the Kihon Checklist (KCL) [28]. The KCL consists of 25 items with 6 subscales, including activities of daily living (5 items), physical ability (5 items), nutrition (2 items), oral condition (3 items), seclusion (2 items), forgetfulness (3 items), and mind (5 items). Life-space mobility will be measured with the LSA [29], which is used to obtain a score based on the reported distance traveled during the 4 weeks preceding assessment. Sleep quality will be evaluated using the Pittsburgh Sleep Quality Index [30], which consists of 19 items that produce a global sleep quality score and scores for 7 components (sleep quality, latency, duration, disturbance, habitual sleep efficiency, use of soporific medication, and daytime dysfunction). Simple nutritional evaluation tools will include the Short Nutritional Assessment Questionnaire [31], which contains 15 items for hospitalized patients and has some items that are most useful for outpatient weight loss, loss of appetite, and use of nutritional supplements and/or tube feeding. Mental health will be measured with the Japanese version of the five-item World Health Organization Well-Being Index, which is a short global rating scale used to measure subjective well-being [32]. The respondent is asked to rate how well each of the five statements applies to him or her when considering the last 14 days. Instrumental activities of daily living evaluation tools will include the Japan Science and Technology Agency Index of Competence (JST-IC) [33]. The JST-IC is a modified version of the Tokyo Metropolitan Institute of Gerontology Index of Competence. Cognitive health will be assessed using subjective memory complaints (SMCs) [34]. SMCs combine the Cambridge Mental Disorders of the Elderly Examination questionnaire and the SMC scale. Hearing screening will be conducted using the Hearing Handicap Inventory for the Elderly–Screening Version (HHIE-S) [35]. The HHIE-S is a ten-item questionnaire developed to assess how an individual perceives the social and emotional effects of hearing loss.

#### Health-related factors

Health-related factors that will be assessed through questionnaires will include blood pressure, medical history, medications, education, smoking status, alcohol intake, whether living alone, outing frequency, self-health status, falls, fall-related injuries, fear of falling, urinary incontinence, habitual exercise, mobility limitations, use of a hearing aid, vision, use of a walking aid, pain, talking, talking time, outdoor self-efficacy, and outdoor social support.

#### Body composition and anthropometry

Body composition and anthropometric measurements will include body weight, and fat and muscle volumes will be measured using multifrequency bioelectrical impedance analysis (MC-980A; TANITA Inc., Tokyo, Japan). Height will also be measured.

### Statistical analysis

The data will be double-entered to enable validation. A coauthor, blinded to group allocation, will conduct preplanned statistical analyses for the primary and secondary outcomes. To ensure blinding, he will have no contact with the participants and use random numbers for group allocation. Intention-to-treat analysis will be applied to the outcome variables. Missing data will be imputed with the multiple imputation method [36]. Characteristics will be compared between groups using chi-square tests for categorical variables and the Mann-Whitney U test for continuous variables. Mixed-model analysis of variance will be carried out to test the effects of group, time, and the group × time interaction for all functional fitness tests. p Values < 0.05 will be considered statistically significant. All statistical analyses will be performed using IBM SPSS Statistics version 24.0 software (IBM, Armonk, NY, USA).

## Discussion

Over one billion people, approximately 15% of the world’s population, are estimated to have some form of disability. These rates are increasing because of an expanding aging population and increases in chronic health conditions such as dementia [37]. Cognitive impairment may be associated with disability in older adults [38]. In this study, we will use a randomized controlled trial design to examine the effectiveness of the KENKOJISEICHI daily outdoor behaviors intervention in community-dwelling older adults with MCI. We hypothesize that participants in the KENKOJISEICHI group will maintain significantly higher cognition at the 6-month follow-up than the educational control group. Based on the transtheoretical model of outdoor behavior change, while cognitive training and cognitive exercise can shift people in the preparation and contemplation stages to the action stage, the outdoor behaviors support program is expected to guide participants to the maintenance stage, which is the essential stage for obtaining enduring health benefits from cognition.

Even during the outdoor behavior support period, older participants may return to an inactive lifestyle because of obstacles such as undesirable weather conditions, health issues, and family and community special events. The outdoor behaviors support program is designed to assist group activity, help them reflect upon issues arising, and help the group come up with its own ideas to overcome such issues. In addition to quantitative assessments of mediating social, psychological, and physiological factors, qualitative assessments focusing on self and group habitual activity will clarify how the outdoor behaviors support program will affect maintaining outdoor behaviors in participants’ daily lives.

If the outdoor behaviors support program successfully promotes maintenance of cognition and physical function, this will expand knowledge regarding supporting change in outdoor behaviors among the older population to obtain health benefits related to cognition. Moreover, the effective and less costly outdoor behaviors programs will be useful in creating a more meaningful and sustainable community-based network.

### Trial status

At the time of manuscript submission, we had obtained ethical approval, registered the trial, and successfully recruited participants. The intervention is in progress (Additional file 1).

## Additional file

Additional file 1: SPIRIT 2013 Checklist: Recommended items to address in a clinical trial protocol and related documents. (DOC 119 kb)

## Abbreviations

GDS: Geriatric Depression Scale
HHIE-S: Hearing Handicap Inventory for the Elderly–Screening Version
ITT: Intention to treat
JST-IC: Japan Science and Technology Agency Index of Competence
KCL: Kihon Checklist
LSA: Life-Space Assessment
MCI: Mild cognitive impairment
MET: Metabolic equivalents
MRI: Magnetic resonance imaging
NCGG-FAT: National Center for Geriatrics and Gerontology-Functional Assessment Tool
PA: Physical activity
SMC: Subjective memory complaints
SPPB: Short Physical Performance Battery
TE: Echo time
TI: Inversion time
TR: Repetition time
